# Transgenerational Shifts in Reproduction Hormesis in Green Peach Aphid Exposed to Low Concentrations of Imidacloprid

**DOI:** 10.1371/journal.pone.0074532

**Published:** 2013-09-06

**Authors:** Murali-Mohan Ayyanath, G. Christopher Cutler, Cynthia D. Scott-Dupree, Paul K. Sibley

**Affiliations:** 1 Department of Environmental Sciences, Faculty of Agriculture, Dalhousie University Agricultural Campus, Truro, Nova Scotia, Canada; 2 School of Environmental Sciences, Ontario Agricultural College, University of Guelph, Guelph, Ontario, Canada; Ghent University, Belgium

## Abstract

Hormesis is a biphasic phenomenon that in toxicology is characterized by low-dose stimulation and high-dose inhibition. It has been observed in a wide range of organisms in response to many chemical stressors, including insects exposed to pesticides, with potential repercussions for agriculture and pest management. To address questions related to the nature of the dose-response and potential consequences on biological fitness, we examined transgenerational hormesis in the green peach aphid, *Myzus persicae,* when exposed to sublethal concentrations of the insecticide imidacloprid. A hormetic response in the form of increased reproduction was consistently observed and a model previously developed to test for hormesis adequately fit some of our data. However, the nature of the dose-response differed within and across generations depending upon the duration and mode of exposure. Decreased reproduction in intermediate generations confirmed that fitness tradeoffs were a consequence of the hormetic response. However, recovery to levels of reproduction equal to that of controls in subsequent generations and significantly greater total reproduction after four generations suggested that biological fitness was increased by exposure to low concentrations of the insecticide, even when insects were continuously exposed to the stressor. This was especially evident in a greenhouse experiment where the instantaneous rate of population increase almost doubled and total aphid production more than quadrupled when aphids were exposed to potato plants systemically treated with low amounts of imidacloprid. Our results show that although fitness tradeoffs do occur with hormetic responses, this does not necessarily compromise overall biological fitness.

## Introduction

Hormesis is a biological phenomenon whereby a stressor can have inhibitory effects at high exposure levels, but at low levels can stimulate biological processes [1], [2]. It has been observed in a plethora of organisms responding to a wide range of chemical, physical, and biological stressors. Oddly, although the consideration of a time component is thought to be critical in understanding hormesis [3], [4], the majority of hormesis studies incorporate only a single time point into their experimental designs [2], [5].

From an evolutionary perspective, only through inclusion of multiple time points in experimental designs can hypotheses related to biological fitness be fully examined. The *principle of allocation* states there are fitness tradeoffs in the allocation of resources among different physiological processes. Increased energy allocation to certain processes (possibly observed as hormesis) is predicted to result in decreased allocation of energy to other processes or traits, and shifts over time in tradeoff expression are expected. Differences in the expression of tradeoffs due to hormesis might vary within and across generations depending on the exposure scenario or duration. However, there is debate as to whether the hormetic response can translate into increased overall fitness, or if there are inherent tradeoffs that render the response to be effectively neutral over the long term [6]–[10]. It has been suggested that in certain situations and for certain species hormesis might be less energetically demanding than expected, and may come at no fitness cost, even if a stressor is not encountered again or encountered at low levels [10].

The consequences of hormetic responses on biological fitness have been observed in invertebrates over multiple generations, with variable results. When exposed to food and temperature stress, *Hydra magnipapillata* Ito asexual reproduction was positively affected without clear tradeoffs, suggesting stresses can have a beneficial impact on fitness-related phenotypical traits in this species [11]. Humic substances that act as mild chemical stressors modified life-history traits of the cladoceran *Moina macrocopa* (Straus), favoring its persistence in fluctuating environments through increased lifespan and promotion of transgenerational resistance to salt stress [12]. When *Daphnia magna* Straus was exposed to low but field-relevant concentrations of fluoxetine, fluvoxamine, and 4-nonylphenol, there was increased offspring production and/or juvenile developmental rates, with different responses depending on life stages and food availability [13]. On the other hand, 21-day exposures of *Daphnia carinata* King to sublethal concentrations of chlorpyrifos resulted in reproductive hormetic effects in the second generation, but reduced reproduction in the first generation and increased pesticide sensitivity in the third generation [14]. Similarly, when the rotifer *Brachionus calyciflorus* Pallas was exposed to low concentrations of dimethoate, increased population growth of the F0 generation was followed by reduced population growth of the F1 and F2 generations, suggesting long-term fitness was compromised by the initial hormetic response [15].

Degradation of pesticides over time and uneven pesticide distribution within the plant canopy make it a virtual certainty that insects will be exposed to low pesticide concentrations in most agricultural fields. Hormesis has been shown to accelerate pest population growth, and pesticide-induced arthropod pest resurgences have been well documented [16]–[22]. Hormesis may also have ramifications for insecticide resistance development [23], and applications for management of beneficial insects [24]–[26]. In the present study we examined the hormetic and transgenerational effects of exposure to sublethal concentrations of imidacloprid on green peach aphid, *Myzus persicae* (Sulzer). *Myzus persicae* is a major worldwide insect pest that is useful in the study of pesticide-induced hormesis in insects [27], [28]. Ecologically it is considered an *r*-selected species that occupies unstable environments (e.g. agro-ecosystems), has high fecundity, and reproduces quickly. We hypothesized that duration and route of exposure to sublethal concentrations of the pesticide would differentially affect the hormetic response in this insect. We expected that continuous exposure to the stressor would result in prolonged hormesis (multiple generations) at lower concentrations, whereas temporary exposure to the stressor would result in short-term hormesis (single generation) at higher concentrations. Through laboratory and greenhouse experiments, effects were studied for up to four generations. We also predicted that although a transgenerational shift in the hormetic response and biological tradeoffs would occur, and might vary with the exposure scenario, there would be no effects on the overall fitness of this *r-*selected species.

## Materials and Methods

### Plant and Insect Maintenance

Potato, *Solanum tuberosum* L. (cv. Kennebec), was grown in 12.5 cm diameter pots containing Pro-Mix® (Halifax Seed, Halifax, Nova Scotia, Canada) potting soil. Plants were watered as needed. Foliage from these plants was used for insect rearing and experiments. *Myzus persicae* was obtained from a wild population infesting broccoli plants (*Brassica oleracea* L.) in a greenhouse at the Faculty of Agriculture, Dalhousie University. Aphid cohorts were maintained on excised leaves in clear plastic boxes (37 L×24 W×14 H cm) lined with deionized water-moistened paper towels. Boxes were held in a growth chamber (22±2°C, 16∶8 L:D, 65±5% RH) and every second day a layer of freshly excised leaves was placed on one end of the box. The infested foliage on the opposite end of the box was discarded when approximately 80% aphids moved to fresh foliage. Paper towels were replaced every 10 days.

### Chemicals

Imidacloprid (Admire® 240 SC, 240 g a.i. L^−1^; Bayer CropScience Canada, Calgary, Alberta, Canada) was suspended in deionized water to obtain a 1000 µg a.i. L^−1^ stock solution. Only the working solutions contained 0.15% TritonX 100 (BDH Chemicals, Toronto, Ontario, Canada) as an emulsifier. Sublethal insecticide concentrations (as determined in preliminary bioassays) of 0.025, 0.1, 0.25, 1.0, 2.5, 10, and 25 µg a.i. L^−1^ were used in leaf-dip exposure experiments, and concentrations of 0.2, 0.6, 2.0, 6.0, 20, 60, and 200 µg a.i. L^−1^ were used in topical exposure experiments. Controls in these experiments consisted of water and 0.15% Triton only. For greenhouse experiments, insecticide solutions of 0 (control), 0.025, 0.1, 0.25, 1.0, 2.5, 10, and 25 µg a.i. L^−1^ were prepared in distilled water. Fresh solutions were prepared for every bioassay replicate.

### Leaf-dip Exposure

Potato leaf discs (1.8 cm diameter) were excised using a stainless steel cork borer. Using forceps, the leaf discs were dipped in control or insecticide solutions for 5 seconds, air-dried for 1 h, and then placed individually in 5.5 cm Petri plates lined with a dry Whatman No. 1 filter paper. In order to avoid cross-contamination, controls were treated first followed by sequential treatment of lowest to highest concentrations of insecticide. Five first instar *M. persicae* (∼ 24 h old) were transferred to each treated leaf disc. Dishes were covered with a Petri plate lid, and placed in sealable plastic containers and held in growth chamber at 22±2°C, 16∶8 L:D, and 65±5% RH.

Depending on the experiment, leaf discs were replaced every second day according to one of two scenarios: a continuous exposure to treated leaf discs where founding first instars were exposed to and reared on treated leaf discs for four generations (G_0_–G_3_); or, a one-time treatment where founding first instars were exposed to treated leaf discs for two days and thereafter reared on untreated leaves for four generations. In all experiments nymph production was recorded every second day. In the continuous exposure experiment, adult aphid length and longevity were also recorded in the first experimental block. The length of 72 h old adults from each generation was measured from the anterior end of the head to the tip of the distal abdominal segment using a microscope and ocular micrometer.

For each exposure scenario, the experiment was a randomized complete block design, with imidacloprid concentration being the main factor of interest. Each bioassay had seven imidacloprid concentrations, and for each there were five Petri dishes with five aphids per dish. Each bioassay was considered an experimental block, and was conducted three times. Repeated measures analyses were conducted using Proc Mixed in SAS [29], with the error terms assumed to be normal with constant variance but not to be independent. Autoregression (AR (1)) represented the appropriate type of dependence for covariance structure. Residuals were used to verify the assumptions of normal error distribution and constant variance. Data were log-transformed as needed to meet the assumptions. If means were significantly different, they were separated using a LSD test (α = 0.05). Backtransformed means are reported as required.

### Topical Exposure

Five first instars *M. persicae* were placed in a clean glass Petri plates (9 cm diameter) and sprayed in a Potter tower (Burkard Scientific, Uxbridge, United Kingdom) at 78 kPa with 2 ml of control or insecticide solution. After each treatment, aphids were transferred to plastic Petri plates (5.5 cm diameter) lined with Whatman No. 1 filter paper containing two untreated potato leaf discs (1.8 cm diameter). Leaf discs were replaced every second day with freshly excised leaf discs. Holding conditions were as described above, except aphids were maintained for two generations only. The experimental design and statistical analysis was as in the leaf-dip exposure experiment.

### Exposure to Systemically Treated Plants

Potatoes were sown in 7.5 cm diameter pots containing Pro-Mix. Approximately two weeks after germination (plant height about 5–7 cm), 50 ml of distilled water or insecticide solution was poured on to the soil surface. Three days later, five first instar *M. persicae* were randomly collected from the stock colony and transferred on to a single randomly selected leaf in the middle of the potato plant. Immediately after transfer of aphids, plants were individually covered with perforated plastic bread bags containing 4–5 holes per cm^−2^ (Prism Pak Inc., Pennsylvania, USA) and secured with an elastic band around the top of the pot. There were three replicates per treatment. Pots were arranged in a completely randomized design in a greenhouse and watered as needed for 21 days. After 21 days in the greenhouse, the total number of aphids per plant was counted.

The greenhouse experiment was repeated three times, and each repetition was considered an experimental block in time. For each potted plant, the instantaneous rate of population increase (*r_i_*) was determined as:
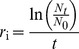
where *N_t_* was the final number of aphids per plant, *N_0_* was the initial number of aphids introduced and *t* was the number of elapsed days during the experiment [30], [31]. Calculated *r_i_* values for each test plant were subjected to mixed model analysis of variance using Proc Mixed followed by a LSD mean separation test (α = 0.05) [29]. Treatment was a fixed factor, and plant and experimental block were considered random factors in the model.

### Dose-Response Modeling

In addition to analysis of variance methods, we used a modified four-parameter logistic model with six parameters developed by Cedergreen et al. [32] to test for hormesis and to assess the dose at which maximal hormetic response occurs. This was done for G_0_ and G_1_ fecundity data of the continuous leaf-dip exposure experiment, and the whole plant greenhouse experiment. The following equation was used:
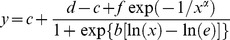
where *d* represents the untreated control; α (0.25) governs the rate at which the hormetic effect manifests; *c* is the lower limit (0) of the dose-response curve; *b* represents the steepness of the curve after the maximal hormetic effect; *e* provides a lower bound on the ED50 level; and *f* measures the rate of stimulation. Parameter *f* cannot be considered a direct representation of the extent of hormesis, but *f>*0 suggests presence of hormesis. The statistical test for the presence of hormesis is represented by analyzing if *f>*0 (*P*<0.1) [32].

Normal distribution and constant variance assumptions on the error terms were verified by examining the residuals of reproductive responses. Data that did not meet these assumptions were square-root transformed 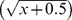 before fitting to the nonlinear model. The dose-response curve with hormetic term *f* was used and all analyses were done using R statistical software with an add-on package drc (http://www.bioassay.dk) [32]. All data were described with models using a lower limit of zero.

## Results

### Leaf-dip Exposure

Exposure to sublethal concentrations of imidacloprid on leaf discs had a significant effect on *M. persicae* fecundity (Table 1). When exposed to treated leaves, the treatment effect was significant in the first three generations but not the fourth, irrespective of whether the treatment exposure was continuous or only for the first two days of the experiment. Except in the foundress generation (G_0_) of the continuous exposure scenario, there was no significant effect of bioassay replicate in any experiment.

**Table 1:** *P*-values for a multigenerational experiment examining effects of imidacloprid concentration and experimental replicate (blocking factor) on *M. persicae* fecundity under laboratory conditions.

Source of Variation	Generation				Total
	G_0_	G_1_	G_2_	G_3_	
Continuous leaf-dip exposure					
Concentration	**0.0001**	**0.0001**	**0.0001**	0.5343	**0.0001**
Bioassay replicate	0.0001	0.0547	0.0710	0.3712	0.7853
One-time leaf-dip exposure					
Concentration	**0.0001**	**0.0001**	**0.0001**	0.4129	**0.0001**
Bioassay replicate	0.2950	0.4101	0.4993	0.1082	0.8371
One-time topical exposure					
Concentration	**0.0001**	**0.0003**	–	–	**0.0001**
Bioassay replicate	0.4758	0.5065	–	–	0.6562

Effects requiring further multiple means comparisons are in bold.

*Continuous Exposure*. When first instars were continuously exposed to treated leaf discs, significant stimulations in fecundity were noted in G_0_ and G_1_ at different concentrations (Table 2). However, these stimulations were absent in G_2_ and G_3_. In this exposure scenario, peak G_0_ reproductive stimulation occurred at 0.025 µg L^−1^ and resulted in a doubling of the number of G_0_ nymphs compared to the controls. In G_1_ the peak hormetic response shifted to a higher concentration of 0.1 µg L^−1^. Continuous exposure to 10 µg L^−1^ of imidacloprid resulted in G_0_ fecundity similar to that seen in the control, reduced fecundity in G_1_ and G_2_, and fecundity similar again to that of the control in G_3_. By the end of the experiment, the total number of progeny produced was greatest in the 0.025 and 0.1 µg L^−1^ treatments, with progeny output in other treatments being equal to or less than that of the control (Table 2).

**Table 2:** Least-squares means of multigenerational fecundity^a^ following continuous exposure of *M. persicae* to sublethal concentrations of imidacloprid.

Concentration(µg L^−1^)	Generation^b^				Total^c^
	G_0_	G_1_	G_2_	G_3_	
0	4.32 bc	4.03 cd	4.01 a	4.43 a	94.52 b
0.025	8.55 a	6.54 ab	1.22 bc	2.27 ab	117.45 a
0.1	5.64 b	8.33 a	1.54 b	2.83 ab	122.14 a
0.25	3.32 cd	2.63 de	1.27 bc	2.53 ab	77.72 bc
1.0	4.10 bc	2.55 e	3.86 a	2.23 ab	90.39 b
2.5	2.82 d	5.30 bc	0.76 cd	3.19 ab	90.90 b
10	3.86 cd	1.82 ef	0.50 d	2.31 ab	63.03 c
25	0.63 e	1.10 f	0.63 d	1.99 b	32.45 d
SEM	0.09	0.12	0.11	0.21	6.68

a 24 h old nymphs were placed on treated potato leaf discs and fecundity of each resulting adult was recorded every 2 days until it died. In the succeeding generation, 5 randomly selected 24 h old nymphs were tracked and fecundity of the resulting adults was recorded every 2 days until they died. G_0_ is initial generation, G_1_ is progeny of G_0_, G_2_ is progeny of G_1_, and G_3_ is progeny of G_2_. Leaf discs were replaced every two days over all generations.

b Progeny per adult data were log transformed before analysis. Backtransformed means are presented. Values followed by different letters are significantly different (LSD, α = 0.05). SEM values are not backtransformed.

c Mean total number of nymphs produced over four generations.

**Figure 1:**
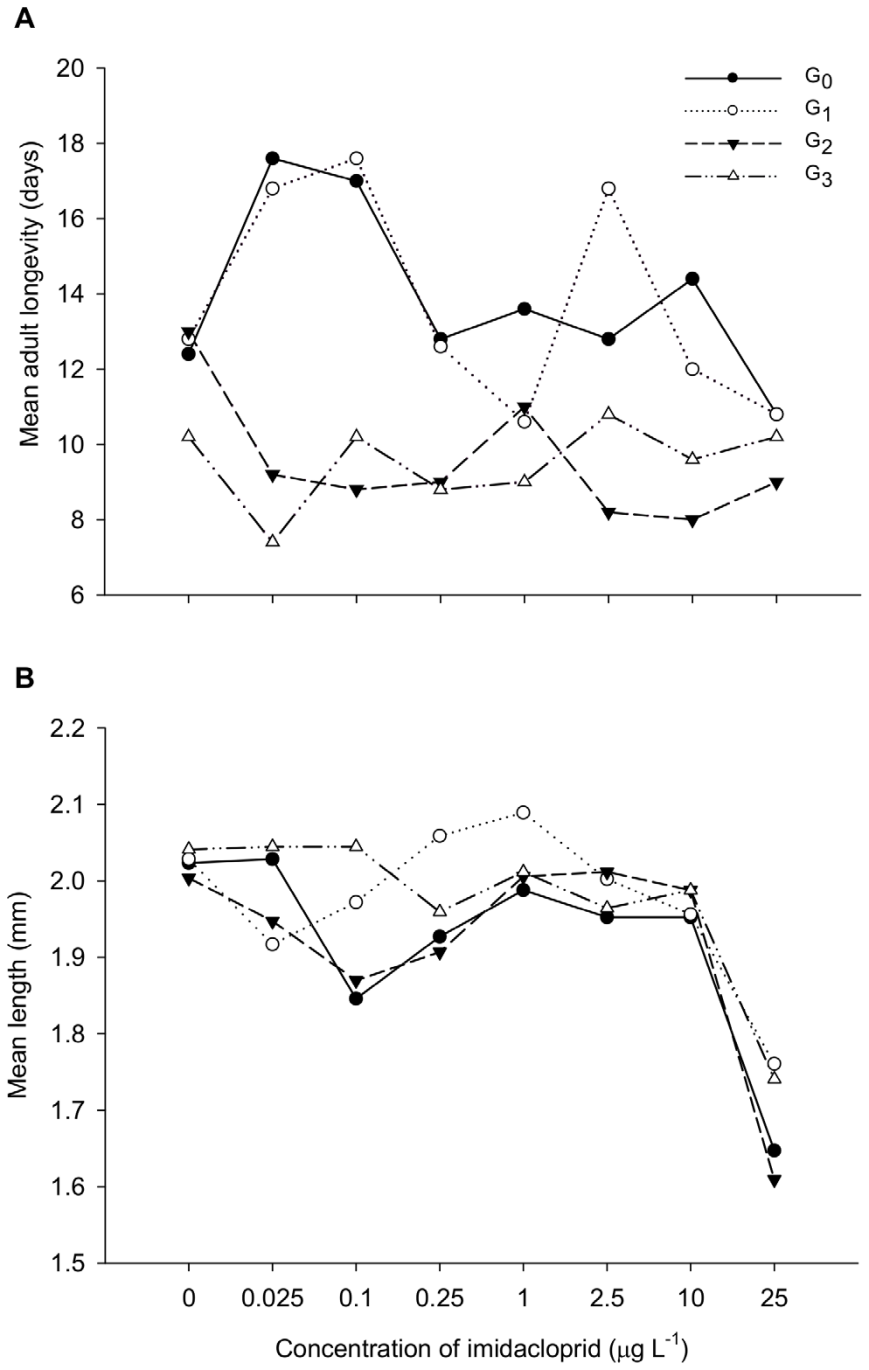
Multigenerational effects of low doses of imidacloprid on aphids. Multigenerational effects of continuous exposure to sublethal concentration of imidacloprid on the (A) longevity and (B) length of adult *M. persicae.*

With continuous exposure, treatment had no effect on aphid longevity except in G_1_ (G_0_: *F_7,32_* = 1.32; *P = *0.27; G_1_: *F_7,32_* = 2.5; *P* = 0.036; G_2_: *F_7,32_* = 1.77; *P = *0.13; G_3_: *F_7,32_* = 0.51; *P = *0.82). However, there were strong differences in adult aphid longevity across generations (*F_3,128_* = 17.58; *P*<0.0001) (Figure 1A). There was no significant treatment-generation interaction on adult longevity (*F_21,128_* = 1.51; *P* = 0.083), but a trend was evident; whereas exposure to concentrations of 0.025 and 0.1 µg L^−1^ tended to increase the longevity of adults above control levels in G_0_ and G_1_, longevity seemed to be reduced at these concentrations in G_2_ and G_3_ (Figure 1A).

With continuous exposure, there were significant treatment effects on the body length of G_0_– G_2_ adults (G_0_: *F_7,42_* = 4.47; *P = *0.0009; G_1_: *F_7,57_* = 2.97; *P = *0.010; G_2_: *F_7,42_* = 3.75; *P = *0.0031) but not G_3_ adults (*F_7,42_* = 0.91; *P* = 0.51). Adults exposed to 25 µg L^−1^ imidacloprid were shorter than controls, but no significant hormetic/stimulatory effect on adult *M. persicae* length was seen at any concentration or generation. There were also significant differences in body length across generations (*F_3,162_* = 2.91; *P* = 0.036) (Figure 1B), but no significant treatment-generation interaction effect on adult body length (*F_21,162_* = 0.98; *P* = 0.49).

*Two-Day Exposure*. In the experiment where only G_0_ foundress aphids were exposed to treated leaf discs for two days, aphid fecundity was stimulated at imidacloprid concentrations of 0.25, 1.0 and 10 µg L^−1^ in G_0_, with 2–3 times more progeny being produced compared to controls (Table 3). In G_1_, only the 0.1 and 1.0 µg L^−1^ treatments had stimulated aphid reproduction, approximately 1.5-fold above that seen in the control. By G_2_, no imidacloprid treatments were stimulatory, and the 0.25 and 10 µg L^−1^ treatments, which were stimulatory in G_0_, gave fewer progeny than the control. Unlike the continuous exposure scenario, the lowest concentration used (0.025 µg L^−1^) resulted in 3-fold lower nymph production than controls in the first generation, and gave the lowest overall fecundity (Table 3). By the end of the experiment, only the 1.0 µg L^−1^ treatment produced more aphids than the control (Table 3). G_0_ nymphs exposed to leaf discs treated with 25 µg L^−1^ imidacloprid did not survive to adulthood.

**Table 3:** Least-squares means of multigenerational fecundity^a^ following two-day exposure of *M. persicae* to sublethal concentrations of imidacloprid.

Concentration(µg L^−1^)	Generation^b^				Total^c^
	G_0_	G_1_	G_2_	G_3_	
0	3.68 de	3.05 b	2.99 a	2.60 a	61.15 b
0.025	1.13 f	1.10 c	1.57 b	1.90 a	38.13 c
0.1	4.54 cd	5.07 a	3.09 a	1.83 a	68.17 b
0.25	6.22 bc	3.52 ab	1.17 b	1.78 a	59.98 b
1.0	8.61 ab	5.30 a	2.78 a	2.44 a	86.88 a
2.5	2.55 e	1.20 c	1.16 b	2.31 a	41.56 c
10^d^	10.52 a	2.56 b	1.43 b	1.61 a	72.89 ab
SEM	0.11	0.13	0.13	0.12	5.33

a 24 h old nymphs were placed on treated potato leaf discs and fecundity of each resulting adult was recorded every 2 days until it died. In the succeeding generation, 5 randomly selected 24 h old nymphs were tracked and fecundity of the resulting adults was recorded every 2 days until they died. G_0_ is initial generation, G_1_ is progeny of G_0_, G_2_ is progeny of G_1_, and G_3_ is progeny of G_2_. G_0_ nymphs were exposed to treated discs for two days and all aphids were thereafter exposed to untreated leaf discs.

b Progeny per adult data were log transformed before analysis. Backtransformed means are presented. Values followed by different letters are significantly different (LSD, α = 0.05). SEM values are not backtransformed.

c Mean total number of nymphs produced over four generations.

d G_0_ nymphs did not survive to adulthood when treated with 25 µg L^−1^. This concentration was not included in the analysis.

### Topical Exposure

Topical exposure to imidacloprid had a significant effect on *M. persicae* reproduction, but there was no difference among bioassay replicates (Table 1). A significant increase in reproduction over the control was found at 0.6 µg L^−1^ in G_0_, but fecundity for all other treatments and time points was equal to or below that of the control (Table 4).

**Table 4:** Least-squares means of two-generational fecundity^a^ following topical exposure of *M. persicae* to sublethal concentrations of imidacloprid.

Concentration (µg L^−1^)	Generation^b^		Total^c^
	G_0_	G_1_	
0	3.65 b	3.37 a	35.02 ab
0.2	1.14 e	1.01 c	12.72 d
0.6	7.69 a	1.09 cd	41.86 a
2	2.01 cde	2.39 ab	23.64 c
6	1.85 de	1.51 bcd	19.71 cd
20	1.41 de	2.36 ab	23.72 c
60	2.40 bcd	2.77 a	27.96 bc
200	3.21 bc	2.04 abc	29.08 bc
SEM	0.13	0.14	3.49

a 24 h old nymphs were topically treated and thereafter reared on untreated potato leaf discs. Fecundity of each resulting adult was recorded every 2 days until it died. In the succeeding generation, 5 randomly selected 24 h old nymphs were tracked and fecundity of the resulting adults was recorded every 2 days until they died. G_0_ is initial generation, G_1_ is progeny of G_0_.

b Progeny per adult data were log transformed before analysis. Backtransformed means are presented. Values followed by different letters are significantly different (LSD, α = 0.05). SEM values are not backtransformed.

c Mean total number of nymphs produced over four generations.

### Exposure to Systemically Treated Plants

The concentrations of imidacloprid applied to soil had a significant effect on *r_i_* (*F_7,62_* = 6.59; *P*<0.0001) and total number of *M. persicae* per plant (*F_7,62_* = 17.28; *P*<0.0001). The difference among experimental blocks was significant for *r_i_* (*F_2,62_* = 3.27; *P = *0.045), but there was no block effect for total number of aphids per plant (*F_2,62_* = 1.95; *P = *0.15). Only the 0.25 µg L^−1^ treatment resulted in a *r_i_* significantly greater than the control, although both the 0.25 and 2.5 µg L^−1^ treatments resulted in significantly more total aphids per plant after 21 days. The 1.0 µg L^−1^ treatment had a significantly lower *r_i_* and fewer total aphids per plant than the control (Table 5).

**Table 5:** Least-squares means of the instantaneous rate of increase (*r_i_*) and total number of aphids per plant after 21 days following infestation of *M. persicae* on to potato plants treated with sublethal concentrations of imidacloprid in a greenhouse.

Concentration (µg L^−1^)	*r_i_* a	Total^a^
0	0.094 bcd	38.53 cd
0.025	0.101 bcd	42.27 bcd
0.1	0.062 de	21.96 de
0.25	0.167 a	173.34 a
1.0	0.034 e	8.93 e
2.5	0.121 ab	72.28 b
10	0.112 bc	54.81 bc
25	0.077 cde	43.30 bcd
SEM	0.016	0.72

a Data were square root transformed before analysis. Backtransformed means are presented. Values followed by different letters are significantly different (LSD, α = 0.05). SEM values are not backtransformed.

In all experiments, depending on the exposure scenario, we found that fecundity outputs at concentrations below or above the hormetic peak concentration, were significantly below those in the control, but increased again at higher concentrations to levels equal to or exceeding the control (Tables 2, 3, 4, 5). We found these effects to be highly reproducible in our experiments.

### Dose-Response Modeling

**Figure 2:**
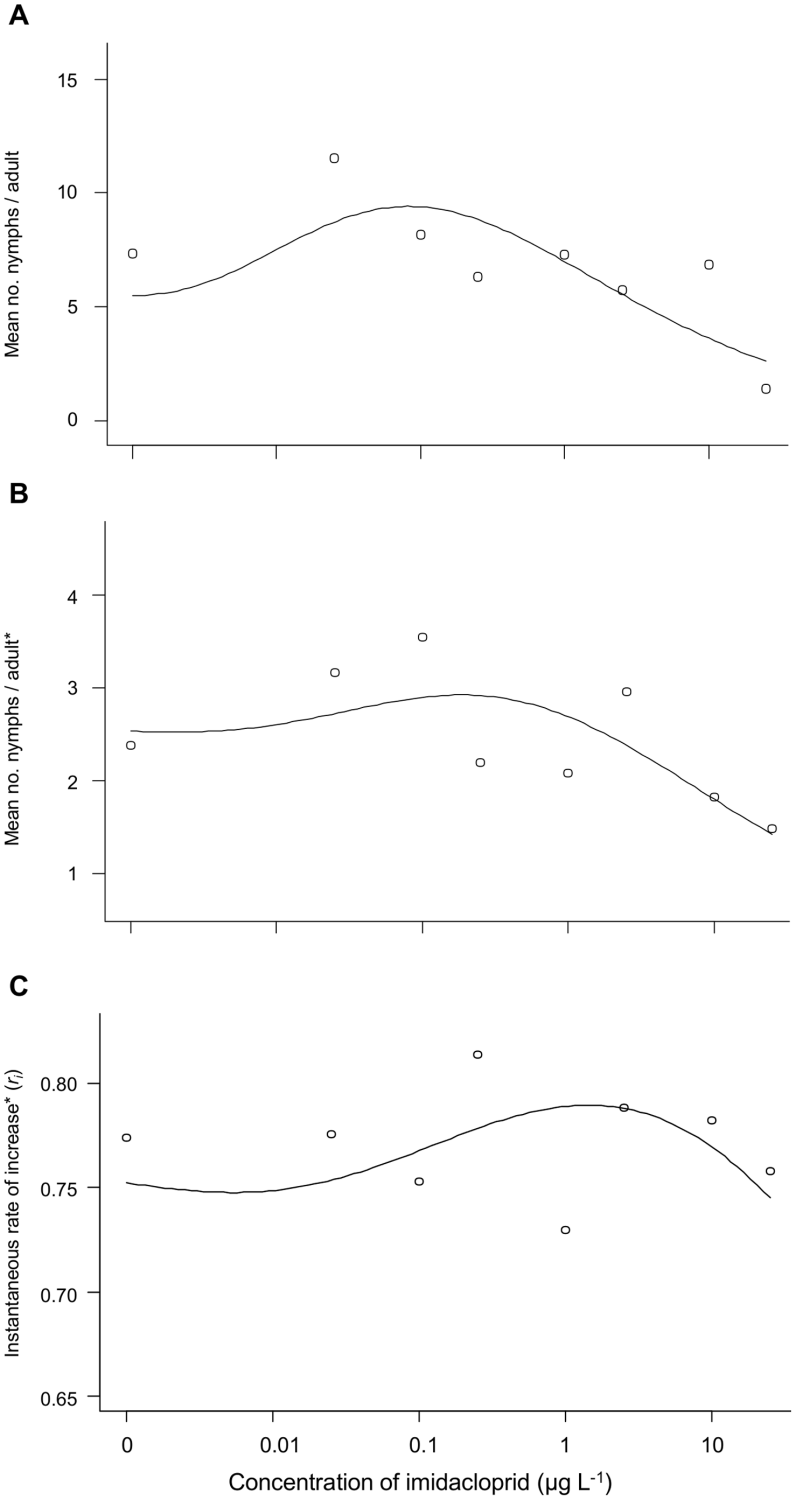
Hormesis model-fitting of low doses of imidacloprid on fecundity and *r_i_* of aphids. Four-parameter biphasic model [32] for reproductive hormetic responses of *M. persicae* in an initial (A) and second (B) generation when continuously exposed to sublethal concentrations of imidacloprid on potato leaf discs, and (C) the instantaneous rate of increase (*r_i_*) of *M. persicae* populations developing on whole potato plants treated with sublethal concentrations of imidacloprid. * indicates data were square-root transformed before analysis.

When *c* was set to zero and α set at 0.25, *f* was significantly different from zero for fecundity of G_0_ (*P*<0.001) and G_1_ (*P* = 0.064) *M. persicae* adults exposed continuously to sublethal concentrations of imidacloprid, and for *r_i_* (*P* = 0.0003) with exposure to potato plants treated with sublethal concentrations of imidacloprid (Table 6). The model found that maximum stimulation (9.42 nymphs per adult) was obtained at 0.08 µg imidacloprid L^−1^ in G_0_, and at 0.18 µg imidacloprid L^−1^ (8.05 nymphs per adult) in G_1_ of aphids. The maximum *r_i_* of 0.123 was obtained at 1.44 µg imidacloprid L^−1^ for *M. persicae* exposed on whole potato plants (Figure 2).

**Table 6:** Regression parameters of model-fitting hormetic responses (G_0_, G_1_ fecundity and *r_i_*) in *M. persicae* exposed to sublethal concentrations of imidacloprid.

Generation	Parameter^a^	Estimate	SE	*t*-value	*P*
**G_0_** b	*b*	0.483	0.031	15.754	0.0000
	*d*	7.721	0.784	9.850	0.0000
	*e*	0.003	0.001	3.852	0.0001
	*f*	327.710	63.221	5.184	0.0000
	RSE	6.064			
	*df*	476			
**G_1_^bc^**	*b*	0.424	0.037	11.451	0.0000
	*d*	2.692	0.194	13.902	0.0000
	*e*	0.421	0.514	0.818	0.4140
	*f*	10.553	5.677	1.859	0.0637
	RSE	1.643			
	*df*	476			
***r_i_*** **^cd^**	*b*	0.238	0.033	7.286	0.0000
	*d*	0.782	0.017	45.954	0.0000
	*e*	672.740	572.848	1.174	0.2466
	*f*	0.475	0.121	3.934	0.0003
	RSE	0.043			
	*df*	44			

a *b,* steepness of the curve after the maximal hormetic effect; *d*, untreated control; *e,* lower bound on the ED50 level; *f,* rate of stimulation; RSE, residual standard error; *df,* degrees of freedom. In the model, *c* was set to zero and α set at 0.25 [32].

b denotes fecundity of *M. persicae* adults continuously exposed to sublethal concentrations of imidacloprid. G_0_ is initial generation, G_1_ is progeny of G_0_.

c data were square-root transformed before analysis.

d *r_i_* is the instantaneous rate of increase of a *M. persicae* population exposed to low-dose imidacloprid treated potato plants.

## Discussion

One of the key questions for scientists that study hormesis within the context of environmental toxicology is, what is the consequence of the hormetic response on biological fitness [6]–[10]? Using the aphid *M. persicae* and insecticide imidacloprid as a model, we implemented various exposure scenarios over multiple generations as a unique approach to examine the temporal nature and biological consequences of the hormetic dose-response. When first instar *M. persicae* were continuously exposed to sublethal concentrations of imidacloprid on leaf discs for four generations, fecundity doubled in the first two generations at certain concentrations, with a shift to a higher peak hormetic concentration from the first to second generation. This was countered by significant reductions in fecundity at the same concentrations in third generation adults, and recovery to fecundity outputs equal to that of controls in the fourth generation_._ This demonstrates that tradeoffs in resource allocation occurred [8], [9], [14], [15].

In an attempt to identify potential intra-generational tradeoffs, aphid length and longevity were recorded for one experimental block in the continuous exposure scenario. No inhibition of longevity or length was found at the hormetic concentrations in the first two generations, and there was a trend (not significant) towards stimulation of longevity. Tradeoffs might have occurred in these initial generations through other phenotypic or physiological traits not measured. Concurrent hormetic responses of multiple traits without obvious biological tradeoffs has similarly been reported in eucalyptus plants exposed to low dose glyphosate [33], and *M. persicae* exposed to low concentrations of imidacloprid [27]. However, we did observe differences among generations with these endpoints, particularly with a tendency towards reduced longevity in generations three and four. This likely reflects a fitness tradeoff experienced due to increased reproductive outputs (hormesis) and energy expenditures in early generations [8], [9].

Despite significantly reduced reproductive outputs and tradeoffs at hormetic concentrations, the total number of aphids at the end of the continuous exposure experiment was significantly greater than that of controls. Total reproductive outputs equal to or exceeding control levels were found at hormetic concentrations in our other experiments as well. This was especially the case in the greenhouse experiment where treatment of plants with 0.25 µg imidacloprid L^−1^ resulted in a significant increase in *r_i_* and 4.5-fold more total aphids than in controls. Previous greenhouse experiments involving sublethal concentrations of imidacloprid and *M. persicae* that did not detect hormesis [34] probably used inappropriate concentrations to detect the effect.

These results suggest there was no long-term fitness cost for the stimulatory response in early generations, supporting one of our hypotheses and results of other multigenerational studies with invertebrates exposed to sublethal amounts of stress [11]–[13]. Although there may be negative energetic consequences (tradeoffs) associated with a hormetic response, energy intake could be slightly increased when an organism is exposed to low levels of a stressor (i.e. in the ‘hormetic zone’) to optimize tradeoffs between self-maintenance and other activities, such as reproduction [10]. The hormetic response might confer a new/adapted normal state that in essence primes or conditions the organism to better cope with higher levels of the stressor when encountered on subsequent occasions [10]–[12]. We speculate that with continued exposure to low levels of the stressor, late generation aphids could better survive imidacloprid exposure than unexposed individuals. This could manifest through induced up-regulation at hormetic concentrations of detoxification enzymes such as esterases [35] or developmental enzymes and proteins [36]. Ultimately, hormetic responses to stress in insects could be a precursor for insecticide resistance development as stress is a general enhancer of mutation rates, which might include mutations leading to pesticide resistance [37].

Dose and temporal patterns of hormesis were somewhat different when founding nymphs were exposed to imidacloprid on leaf discs for only 2 days. At the lowest concentration of 0.025 µg L^−1^, intra-generational and overall reproduction was well below that of the control. Low reproduction at the lowest concentration was also seen in the topical exposure and greenhouse experiments, and we have previously observed this in continuous exposure leaf-dip experiments (M-M.A. unpublished data). High first generation fecundity at 0.025 µg L^−1^ under continuous exposure resulted in a doubling in reproductive output when insects reached adulthood. In contrast, with the shorter 2-day or topical exposure, by the time 0.025 µg L^−1^ treated nymphs reached adulthood there was no longer any exposure to the insecticide and the hormetic response to the stressor was complete. Thereafter, the insects entered the tradeoff phase of the response, represented by lower reproductive output. This supports our prediction that short-term exposure to the stressor would result in short-term hormesis expressed at higher concentrations relative to that seen with continuous exposure to the stressor.

Hormetic responses occurred at several concentrations in aphids of the first two generations following the 2-day exposure, at concentrations higher than in the continuous exposure, again reflecting what we believe is the insect’s ability to better tolerate higher concentrations when exposed for shorter periods. The occurrence of pre-hormetic toxicity (reduced fecundity) that we observed with short-term exposure has previously been observed in insects [38] and in plants [39]. In our experiments, at the lowest imidacloprid concentration, nymphs might have been able to allocate an adequate amount of resource towards coping with the stress before reaching adulthood. If this were the case, by the time these nymphs reached adulthood, they would have been in the tradeoff phase of the response, observed as lowered reproduction in adults. This was not seen in the continuous exposure scenario because aphids were coping with the stressor right up to adulthood, and the tradeoff was not observed until subsequent generations following hormesis. With short-term exposure at higher concentrations, nymphs required more resources and time to cope with the stress, resulting in hormesis and higher reproduction in first generation adults. Low-dose stimulatory effects seen as hormesis are likely not the only toxicological phenomenon occurring in the low dose range [17], [39], [40].

The logistic model developed by Cedergreen et al. [32] detected reproductive hormesis in first and second generation aphids exposed continuously to imidacloprid on leaf discs, and in *r_i_* from our greenhouse experiment, corroborating our analysis of variance and population growth analyses. The predicted hormetic peak was slightly lower than that found in our experiments. This is probably partially because the model requires 4–5 hormetic doses to adequately describe the hormetic response [32], whereas our data allowed for only 1–2 hormetic doses. Although the model provides a significant fit for hormesis, we feel it masks certain results in some of the data. The current model does not adequately address pre-hormetic toxicity that we found in several of our experiments, a limitation that has previously been noted in statistical models that describe hormesis [39]. The model also does not adequately highlight the sharp “dips” in the dose-response that we consistently detected after the hormetic peak in several exposure scenarios. The occurrence and mechanisms of this phenomenon should be explored further, along with refinement of models that better take into account such pre-hormetic toxicity.

In conclusion, the hormetic response in *M. persicae* exposed to low doses of imidacloprid was robust and highly reproducible. However, intra- and transgenerational reproductive responses differed depending on the exposure scenario. Despite tradeoffs in transgenerational reproduction, this did not adversely affect total reproductive output after four generations, suggesting that overall fitness was not adversely affected. In some situations fitness tradeoffs due to hormesis may render the phenomenon evolutionary neutral [8], [9], and our results show that hormetic responses need not come at the expense of biological fitness. Indeed, hormesis is likely a critical adaptation for organisms that allows them to adjust to fluctuations in their environment, possibly acting as a ‘conditioning’ mechanism that enables the organism to better cope with subsequent exposure to higher levels of the stressor [41]. Costantini et al. [10] suggested that in certain situations such conditioning could increase biological fitness, even if the stressor was not encountered again, or encountered at low levels. As we predicted, this would seem particularly important for *r-*selected species that are specialized for high and rapid reproduction in unstable and unpredictable environments. Insect pests that are targets of frequent pesticide applications in agroecosystems clearly represent such a scenario.
